# 3,3′-Bis(3-meth­oxy­benz­yl)-1,1′-(ethane-1,2-diyl)­diimidazolium dibromide dihydrate

**DOI:** 10.1107/S1600536811050240

**Published:** 2011-11-30

**Authors:** Hon Man Lee, Pi-Yun Cheng

**Affiliations:** a Department of Chemistry, National Changhua University of Education, Changhua, Taiwan 50058

## Abstract

In the title compound, C_24_H_28_N_4_O_2_^2+^·2Br^−^·2H_2_O, the diimid­azo­lium cation is located on an inversion center. The imidazole and the benzene rings make a dihedral angle of 68.08 (04)°. In the crystal, O—H⋯Br, C—H⋯O and C—H⋯Br hydrogen bonds link the diimidazolium cations, the bromide anions and the water mol­ecules into a two-dimensional network.

## Related literature

For the non-hydrated crystal structure of the title compound, see: Lee & Lu (2008 ▶). For preparation of the title compound, see: Lee *et al.* (2004 ▶).
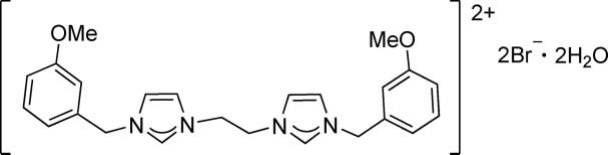


## Experimental

### 

#### Crystal data

C_24_H_28_N_4_O_2_^2+^·2Br^−^·2H_2_O
*M* *_r_* = 600.36
Monoclinic, 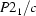

*a* = 8.5879 (3) Å
*b* = 13.7822 (6) Å
*c* = 11.0964 (5) Å
β = 107.277 (2)°
*V* = 1254.11 (9) Å^3^
*Z* = 2
Mo *K*α radiation
μ = 3.27 mm^−1^
*T* = 150 K
0.25 × 0.20 × 0.20 mm

#### Data collection

Bruker SMART APEXII diffractometer
Absorption correction: multi-scan (*SADABS*; Sheldrick, 2003 ▶) *T*_min_ = 0.495, *T*_max_ = 0.561
9596 measured reflections
2986 independent reflections
2560 reflections with *I* > 2σ
*R*_int_ = 0.020

#### Refinement

*R*[*F* ^2^ > 2σ(*F* ^2^)] = 0.023
*wR*(*F* ^2^) = 0.059
*S* = 1.04
2986 reflections
154 parameters
H-atom parameters constrained
Δρ_max_ = 0.34 e Å^−3^
Δρ_min_ = −0.26 e Å^−3^

### 

Data collection: *APEX2* (Bruker, 2007 ▶); cell refinement: *SAINT* (Bruker, 2007 ▶); data reduction: *SAINT*; program(s) used to solve structure: *SHELXS96* (Sheldrick, 2008 ▶); program(s) used to refine structure: *SHELXTL* (Sheldrick, 2008 ▶); molecular graphics: *SHELXTL*; software used to prepare material for publication: *DIAMOND* (Brandenburg, 2006 ▶).

## Supplementary Material

Crystal structure: contains datablock(s) I, global. DOI: 10.1107/S1600536811050240/pv2485sup1.cif

Structure factors: contains datablock(s) I. DOI: 10.1107/S1600536811050240/pv2485Isup2.hkl

Supplementary material file. DOI: 10.1107/S1600536811050240/pv2485Isup3.cml

Additional supplementary materials:  crystallographic information; 3D view; checkCIF report

## Figures and Tables

**Table 1:** Hydrogen-bond geometry (Å, °)

*D*—H⋯*A*	*D*—H	H⋯*A*	*D*⋯*A*	*D*—H⋯*A*
O2—H12⋯Br1^i^	0.74	2.63	3.3419 (13)	161.9
O2—H13⋯Br1	0.89	2.52	3.3362 (14)	153.2
C11—H1⋯O2^ii^	0.91	2.45	3.295 (2)	154.5
C1—H6⋯Br1^ii^	0.94	2.86	3.6743 (16)	145.5
C5—H4⋯O2^ii^	0.95	2.60	3.402 (2)	142.6

Symmetry codes: (i) 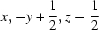; (ii) 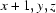.

**Fig. 1.**
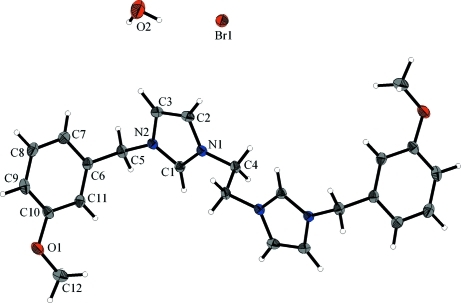
The structure of the title compound, showing 50% probability displacement ellipsoids for the non-hydrogen atoms. The H atoms are dipicted by circles of an arbitrary radius. The unlabelled atoms of the imidazolium cation are related to the labelled ones by symmetry operation: 2 - x, -y, 1 - z; for the anion, the symmetry operation for Br1 is x, 1/2 - y, -1/2 + z.

**Fig. 2.**
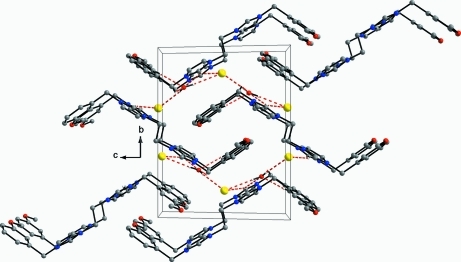
Unit cell packing of the title compound showing hydrogen bonding interactions.

## supplementary crystallographic information

### Comment

The non-hydrated crystal structure of the same imidazolium bromide, has been published previously (Lee & Lu, 2008). The title compound is a dihydrated salt. The structure of the title compound (Fig. 1) is similar to the non-hydrated structure, wherein the bis(imidazolium) dication is also located on an inversion center with the two imidazole rings being parallel to each other. The imidazole and the benzene rings in the title compound make a dihedral angle of 68.08 (04)°, which is much smaller than 77.25 (16)° reported in the non-hydrated structure. Intermolecular hydrogen bonds of the type O—H···Br, C—H···O and C—H···Br link the imidazolium cations, bromide anions and water solvent molecules, forming a two-dimensional hydrogen-bonded network (Fig. 2 and Table 1).

### Experimental

The compound was prepared according to the literature procedure (Lee *et al.*, 2004). Suitable crystals were obtained by slow diffusion of diethyl ether into a wet DMF solution of the compound at room temperature.

### Refinement

The methyl hydrogen atoms were positioned geometrically and refined as riding atoms, with C—H = 0.98 Å with *U*_iso_(H) = 1.5*U*_eq_(C). The other hydrogen atoms are located from the difference Fourier maps, allowed to refine in the initial refinement cycles and then fixed at those positions during the final cycles of refinement.

### Crystal data

**Table:**

C_24_H_28_N_4_O_2_^2+^·2Br^−^·2H_2_O	*F*(000) = 612
*M**_r_* = 600.36	*D*_x_ = 1.590 Mg m^−^^3^
Monoclinic, *P*2_1_/*c*	Mo *K*α radiation, λ = 0.71073 Å
Hall symbol: -P 2ybc	Cell parameters from 4076 reflections
*a* = 8.5879 (3) Å	θ = 2.4–27.8°
*b* = 13.7822 (6) Å	µ = 3.27 mm^−^^1^
*c* = 11.0964 (5) Å	*T* = 150 K
β = 107.277 (2)°	Block, white
*V* = 1254.11 (9) Å^3^	0.25 × 0.20 × 0.20 mm
*Z* = 2	

### Data collection

**Table:**

Bruker SMART APEXII diffractometer	2986 independent reflections
Radiation source: fine-focus sealed tube	2560 reflections with *I* > 2σ
graphite	*R*_int_ = 0.020
ω scans	θ_max_ = 27.9°, θ_min_ = 2.4°
Absorption correction: multi-scan (*SADABS*; Sheldrick, 2003)	*h* = −11→11
*T*_min_ = 0.495, *T*_max_ = 0.561	*k* = −18→17
9596 measured reflections	*l* = −10→14

### Refinement

**Table:**

Refinement on *F*^2^	Primary atom site location: structure-invariant direct methods
Least-squares matrix: full	Secondary atom site location: difference Fourier map
*R*[*F*^2^ > 2σ(*F*^2^)] = 0.023	Hydrogen site location: inferred from neighbouring sites
*wR*(*F*^2^) = 0.059	H-atom parameters constrained
*S* = 1.04	*w* = 1/[σ^2^(*F*_o_^2^) + (0.0332*P*)^2^ + 0.2216*P*] where *P* = (*F*_o_^2^ + 2*F*_c_^2^)/3
2986 reflections	(Δ/σ)_max_ = 0.004
154 parameters	Δρ_max_ = 0.34 e Å^−^^3^
0 restraints	Δρ_min_ = −0.26 e Å^−^^3^

### Special details

**Table:**

Geometry. All e.s.d.'s (except the e.s.d. in the dihedral angle between two l.s. planes) are estimated using the full covariance matrix. The cell e.s.d.'s are taken into account individually in the estimation of e.s.d.'s in distances, angles and torsion angles; correlations between e.s.d.'s in cell parameters are only used when they are defined by crystal symmetry. An approximate (isotropic) treatment of cell e.s.d.'s is used for estimating e.s.d.'s involving l.s. planes.
Refinement. Refinement of *F*^2^ against ALL reflections. The weighted *R*-factor *wR* and goodness of fit *S* are based on *F*^2^, conventional *R*-factors *R* are based on *F*, with *F* set to zero for negative *F*^2^. The threshold expression of *F*^2^ > σ(*F*^2^) is used only for calculating *R*-factors(gt) *etc*. and is not relevant to the choice of reflections for refinement. *R*-factors based on *F*^2^ are statistically about twice as large as those based on *F*, and *R*- factors based on ALL data will be even larger.

### Fractional atomic coordinates and isotropic or equivalent isotropic displacement parameters (Å^2^)

**Table:**

	*x*	*y*	*z*	*U*_iso_*/*U*_eq_	
Br1	0.359161 (19)	0.146752 (12)	0.490121 (15)	0.02394 (7)	
C1	0.92203 (19)	0.14465 (11)	0.31701 (14)	0.0160 (3)	
C11	0.9179 (2)	0.13818 (11)	−0.03275 (15)	0.0177 (3)	
C5	0.8031 (2)	0.23349 (11)	0.11436 (14)	0.0184 (3)	
C3	0.6531 (2)	0.14528 (12)	0.24893 (16)	0.0209 (3)	
C7	0.6246 (2)	0.14644 (12)	−0.07555 (16)	0.0222 (3)	
C6	0.7804 (2)	0.17085 (11)	−0.00127 (14)	0.0168 (3)	
C10	0.8960 (2)	0.08036 (12)	−0.13955 (14)	0.0193 (3)	
C4	0.96949 (19)	0.04905 (12)	0.51517 (14)	0.0180 (3)	
C8	0.6059 (2)	0.08805 (13)	−0.18098 (15)	0.0236 (4)	
C2	0.69825 (19)	0.09468 (12)	0.35762 (15)	0.0204 (3)	
C9	0.7400 (2)	0.05481 (12)	−0.21304 (14)	0.0225 (4)	
N1	0.86714 (16)	0.09507 (9)	0.39930 (11)	0.0157 (3)	
N2	0.79482 (16)	0.17629 (10)	0.22518 (12)	0.0154 (3)	
O1	1.02188 (14)	0.04640 (9)	−0.18024 (11)	0.0257 (3)	
O2	0.21749 (17)	0.23175 (12)	0.19624 (13)	0.0436 (4)	
C12	1.1839 (2)	0.07413 (14)	−0.10897 (17)	0.0264 (4)	
H12A	1.2623	0.0455	−0.1472	0.040*	
H12B	1.2066	0.0509	−0.0220	0.040*	
H12C	1.1934	0.1450	−0.1089	0.040*	
H1	1.0194	0.1540	0.0159	0.020*	
H3	0.5023	0.0720	−0.2314	0.021*	
H2	0.5349	0.1704	−0.0595	0.026*	
H5	0.7189	0.2815	0.1019	0.015*	
H4	0.9057	0.2648	0.1384	0.016*	
H6	1.0329	0.1567	0.3255	0.017*	
H7	0.5462	0.1622	0.1957	0.029*	
H8	1.0586	0.0915	0.5535	0.025*	
H9	0.9081	0.0408	0.5698	0.023*	
H12	0.2685	0.2563	0.1623	0.059*	
H13	0.2877	0.2129	0.2690	0.062*	
H15	0.7253	0.0152	−0.2863	0.031*	
H16	0.6386	0.0624	0.4035	0.025*	

### Atomic displacement parameters (Å^2^)

**Table:**

	*U*^11^	*U*^22^	*U*^33^	*U*^12^	*U*^13^	*U*^23^
Br1	0.02082 (10)	0.02866 (11)	0.02254 (10)	−0.00226 (7)	0.00672 (7)	0.00185 (7)
C1	0.0177 (7)	0.0153 (8)	0.0149 (7)	0.0004 (6)	0.0048 (6)	−0.0012 (6)
C11	0.0196 (8)	0.0171 (8)	0.0155 (7)	−0.0023 (6)	0.0040 (6)	0.0015 (6)
C5	0.0249 (8)	0.0143 (8)	0.0164 (7)	0.0018 (7)	0.0068 (6)	0.0018 (6)
C3	0.0155 (7)	0.0257 (9)	0.0216 (8)	0.0017 (7)	0.0058 (6)	−0.0015 (7)
C7	0.0215 (8)	0.0244 (9)	0.0199 (8)	0.0040 (7)	0.0047 (7)	0.0021 (7)
C6	0.0216 (8)	0.0133 (8)	0.0148 (7)	0.0015 (6)	0.0042 (6)	0.0021 (6)
C10	0.0261 (8)	0.0171 (8)	0.0170 (7)	0.0003 (7)	0.0102 (7)	0.0027 (6)
C4	0.0223 (8)	0.0192 (8)	0.0129 (7)	0.0012 (7)	0.0058 (6)	0.0009 (6)
C8	0.0218 (8)	0.0267 (9)	0.0188 (8)	−0.0036 (7)	0.0005 (6)	0.0009 (7)
C2	0.0178 (8)	0.0220 (9)	0.0239 (8)	−0.0009 (7)	0.0101 (7)	−0.0004 (7)
C9	0.0317 (9)	0.0204 (9)	0.0153 (7)	−0.0060 (7)	0.0069 (7)	−0.0026 (6)
N1	0.0182 (6)	0.0145 (7)	0.0156 (6)	0.0010 (5)	0.0068 (5)	0.0001 (5)
N2	0.0172 (6)	0.0141 (6)	0.0157 (6)	0.0009 (5)	0.0063 (5)	−0.0006 (5)
O1	0.0270 (6)	0.0312 (7)	0.0227 (6)	−0.0013 (5)	0.0129 (5)	−0.0059 (5)
O2	0.0347 (8)	0.0646 (11)	0.0281 (7)	−0.0162 (7)	0.0040 (6)	0.0061 (7)
C12	0.0248 (9)	0.0262 (9)	0.0319 (9)	−0.0004 (7)	0.0142 (7)	−0.0004 (7)

### Geometric parameters (Å, °)

**Table:**

C1—N2	1.327 (2)	C10—C9	1.391 (2)
C1—N1	1.3337 (19)	C4—N1	1.4686 (19)
C1—H6	0.9430	C4—C4^i^	1.523 (3)
C11—C10	1.394 (2)	C4—H8	0.9571
C11—C6	1.401 (2)	C4—H9	0.9209
C11—H1	0.9051	C8—C9	1.381 (2)
C5—N2	1.4800 (19)	C8—H3	0.9264
C5—C6	1.510 (2)	C2—N1	1.385 (2)
C5—H5	0.9596	C2—H16	0.9355
C5—H4	0.9459	C9—H15	0.9552
C3—C2	1.347 (2)	O1—C12	1.433 (2)
C3—N2	1.387 (2)	O2—H12	0.7395
C3—H7	0.9616	O2—H13	0.8910
C7—C6	1.388 (2)	C12—H12A	0.9800
C7—C8	1.389 (2)	C12—H12B	0.9800
C7—H2	0.9033	C12—H12C	0.9800
C10—O1	1.3720 (19)		
			
N2—C1—N1	108.44 (13)	N1—C4—H9	108.6
N2—C1—H6	126.5	C4^i^—C4—H9	109.7
N1—C1—H6	125.0	H8—C4—H9	108.8
C10—C11—C6	118.97 (15)	C9—C8—C7	120.81 (15)
C10—C11—H1	120.5	C9—C8—H3	119.4
C6—C11—H1	120.5	C7—C8—H3	119.8
N2—C5—C6	112.06 (13)	C3—C2—N1	106.92 (13)
N2—C5—H5	105.5	C3—C2—H16	132.5
C6—C5—H5	111.7	N1—C2—H16	120.6
N2—C5—H4	106.2	C8—C9—C10	119.78 (15)
C6—C5—H4	112.0	C8—C9—H15	119.9
H5—C5—H4	109.0	C10—C9—H15	120.3
C2—C3—N2	107.13 (14)	C1—N1—C2	108.74 (13)
C2—C3—H7	130.1	C1—N1—C4	125.41 (13)
N2—C3—H7	122.7	C2—N1—C4	125.85 (13)
C6—C7—C8	119.38 (15)	C1—N2—C3	108.76 (13)
C6—C7—H2	121.5	C1—N2—C5	125.55 (13)
C8—C7—H2	119.0	C3—N2—C5	125.68 (13)
C7—C6—C11	120.59 (15)	C10—O1—C12	117.28 (12)
C7—C6—C5	120.07 (14)	H12—O2—H13	104.6
C11—C6—C5	119.34 (15)	O1—C12—H12A	109.5
O1—C10—C9	115.89 (14)	O1—C12—H12B	109.5
O1—C10—C11	123.64 (15)	H12A—C12—H12B	109.5
C9—C10—C11	120.47 (15)	O1—C12—H12C	109.5
N1—C4—C4^i^	110.22 (15)	H12A—C12—H12C	109.5
N1—C4—H8	108.5	H12B—C12—H12C	109.5
C4^i^—C4—H8	111.0		

Symmetry codes: (i) −*x*+2, −*y*, −*z*+1.

### Hydrogen-bond geometry (Å, °)

**Table:**

*D*—H···*A*	*D*—H	H···*A*	*D*···*A*	*D*—H···*A*
O2—H12···Br1^ii^	0.74	2.63	3.3419 (13)	161.9
O2—H13···Br1	0.89	2.52	3.3362 (14)	153.2
C11—H1···O2^iii^	0.91	2.45	3.295 (2)	154.5
C1—H6···Br1^iii^	0.94	2.86	3.6743 (16)	145.5
C5—H4···O2^iii^	0.95	2.60	3.402 (2)	142.6

Symmetry codes: (ii) *x*, −*y*+1/2, *z*−1/2; (iii) *x*+1, *y*, *z*.

## References

[bb1] Brandenburg, K. (2006). *DIAMOND* Crystal Impact GbR, Bonn, Germany.

[bb2] Bruker (2007). *APEX2* and *SAINT* Bruker AXS Inc., Madison, Wisconsin, USA.

[bb3] Lee, H. M. & Lu, C.-Y. (2008). *Acta Cryst.* E**64**, o2086. 10.1107/S1600536808031863 PMC2959526 21580951

[bb4] Lee, H. M., Lu, C. Y., Chen, C. Y., Chen, W. L., Lin, H. C., Chiu, P. L. & Cheng, P. Y. (2004). *Tetrahedron*, **60**, 5807–5825.

[bb5] Sheldrick, G. M. (2003). *SADABS* University of Göttingen, Germany.

[bb6] Sheldrick, G. M. (2008). *Acta Cryst.* A**64**, 112–122. 10.1107/S0108767307043930 18156677

